# Management of an Unusual Orbitocranial Penetrating Injury

**DOI:** 10.1155/2020/9070595

**Published:** 2020-02-14

**Authors:** Han Y. Yin, Swetha Dhanireddy, Alexander E. Braley

**Affiliations:** ^1^SUNY Upstate Medical University, Department of Ophthalmology and Visual Sciences, Syracuse, NY, USA; ^2^SUNY Upstate Medical University, Department of Neurosurgery, Syracuse, NY, USA; ^3^Florida International University Herbert Wertheim College of Medicine, Miami, FL, USA

## Abstract

**Background:**

An intraorbital injury with a blunt penetrating intraorbital foreign body (IOFB) is an unusual cause of penetrating trauma. This type of trauma is considered a surgical emergency given the risk to vision in addition to potential intracranial injuries such as vascular injury, dural laceration, and neurologic injury. A thorough history and physical exam, along with careful radiographic and multidiscipline intervention, is crucial in providing the patient the most appropriate care. *Case Presentation*. A 66-year-old male presented to the emergency room (ER) after falling down the stairs and suffering an orbitocranial penetrating injury. He underwent urgent fluoroscopy-guided foreign body removal with a multidisciplinary team after a workup revealed no significant ocular or intracranial injuries. The foreign body was removed with an anterior approach without any complications.

**Conclusion:**

In this study, we demonstrated that IOFB in proximity to orbitocranial structures requires a careful multidisciplinary team approach. An interventional radiology- (IR-) guided approach in extracting the foreign body is essential to prevent further injury. A high dose of intravenous steroid was not used due to initial suspicion of intracranial involvement. Prompt removal decreased risk of further vision loss.

## 1. Background

Penetrating orbitocranial injury carries high mortality and morbidity. Although it is rare, a retained intraorbital foreign body (IOFB) is often a result of high-velocity projectile trauma with a sharp-tipped object. Often, the integrity of orbital contents is violated, resulting in ruptured globe, vascular injury, lid laceration, and orbital bone fractures [1, 2]. Furthermore, deep orbital structures and adjacent intracranial structures are at risk of being injured from a penetrating foreign body. Clearly all intraorbital vascular and neural structures are at risk during intraocular trauma, but trauma that violates the orbital wall makes nearby intracranial structures vulnerable, with potentially catastrophic sequelae. Such structures include but are not limited to the internal carotid artery (ICA), basilar artery, cavernous sinus, pituitary gland, and cranial nerves I-VI, in addition to the frontal lobe, temporal lobe, and the brain stem. Therefore, immediate evaluation and appropriate surgical management are critical. Trauma with IOFB adjacent to the optic nerve is considered an ophthalmologic emergency.

Here, we present a rare and unusual case of facial trauma resulting from a low-velocity injury with a blunt tipped object. This injury led to IOFB with close proximity to adjacent intracranial tissue, therefore warranting an early multidisciplinary surgical intervention to conquer a unique and challenging surgical case.

## 2. Case Presentation

**Figure 1:**
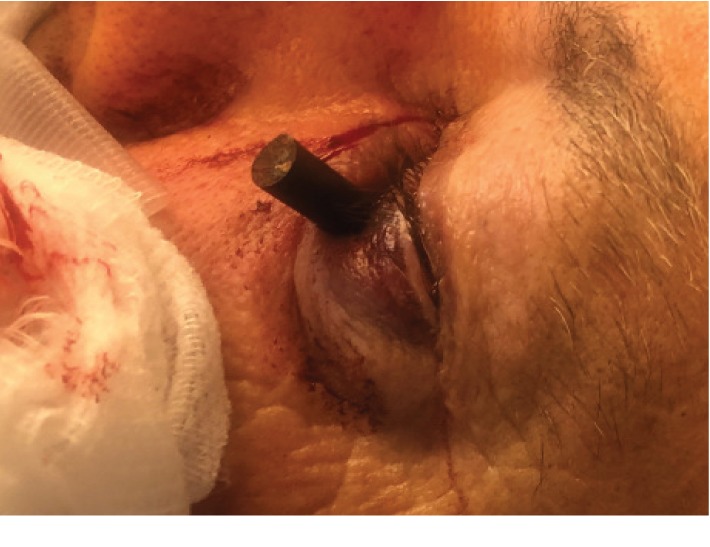
External photograph of the patient's left eye, with a metallic foreign body extruding out from the left lower lid above the inferior orbital rim.

A 66-year-old right-handed male presented to the emergency room with orbitocranial penetrating injury after falling down the stairs. During the fall, he impacted his face on the coat rack at the bottom of the stairs. One of the coat hooks on the coat rack penetrated into his left orbit and lodged into the posterior orbital wall. The coat hook was cut in the field by the paramedics two to three centimeters from the point of entry. On presentation, he was alert and fully oriented with a Glasgow Coma Scale (GCS) of 15 and his neurological exam was nonfocal. His ophthalmologic exam revealed an edematous left lower eyelid, with a visible large metallic foreign body breached through the lower eyelid bordering the inferior orbital rim (Figure 1). The visual acuity in his left eye (OS) was 20/200 with no improvement on the pinhole, and intraocular pressure (IOP) assessment was deferred in the left eye. There was a left relative afferent pupillary defect (RAPD). Ocular movements were intact in both eyes. The subsequent ophthalmic exam did not suggest an occult globe rupture.

**Figure 2:**
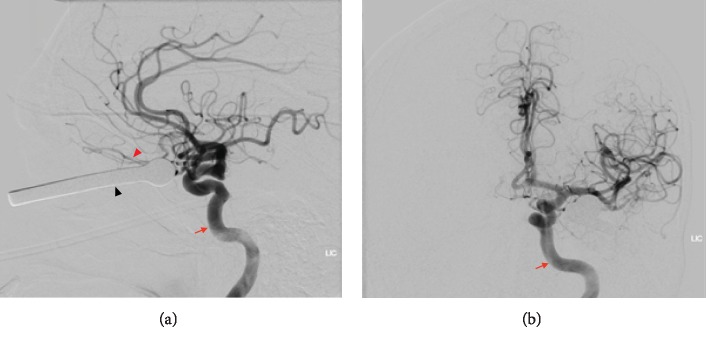
(a) Lateral and (b) Towne's view of an arterial phase digital subtraction angiography showing left internal carotid artery injection demonstrating no major arterial injury. The subtracted silhouette of the foreign object still protruding from the patient's left eye is also visualized.

**Figure 3:**
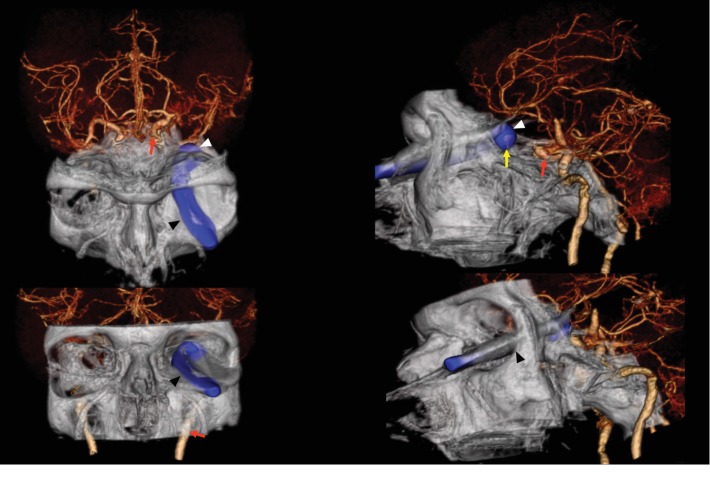
CT angiography with a 3D reconstructed image, demonstrating orbitocranial junction and adjacent arterial vasculature with no apparent vascular injury. The black arrowhead depicts IOFB, the white arrowhead shows the blunt tip of IOFB, the red arrow shows the internal carotid artery, and the yellow arrow shows the comminuted sphenoid bone fracture.

Computed tomography (CT) head was obtained and did not appear to show any significant intracranial injury, although the cast iron hook created a significant metal artifact that limited the quality of the study. CT angiography (CTA) did not show any vascular injury but was also severely limited by the metal streak artifact. He was started on meningitis prophylaxis, and a tetanus booster was administered. He was taken for an urgent diagnostic cerebral angiogram to better investigate the intracranial vascular structures prior to any attempts to remove the hook. This was critical as the metal streak artifact may have obscured an underlying vascular injury that could complicate hook removal. Six-vessel cerebral angiography did not show injury to any intracranial vessels (Figure 2). The majority of the intraorbital vessels appeared uninjured, although the left superior ophthalmic vein was not visualized; this was concluded to be secondary to compression from the foreign object or possibly from thrombosis. A reconstructed 3-dimensional (3D) image confirmed that there was no major violation of vascular structures (Figure 3).

**Figure 4:**
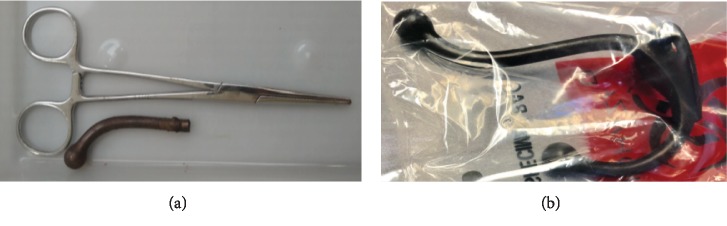
(a) Foreign body after transorbital removal. (b) Another coat hook from the same rack provided to medical personnel by the patient's family.

The patient underwent an orbital foreign body removal under fluoroscopic guidance in a multidisciplinary setting with neurosurgery, ophthalmology, and neurointerventional radiology. The neurointerventional radiologist was in the room for immediate support for potential vascular injuries. The groin and the orbit were prepped and draped in the usual sterile fashion. The hook was rotated with ease, but initial resistance to withdrawal of the hook suggested that there may have been a ball-socket interaction with the orbital wall and the ball of the hook, which had to be overcome. The hook then was released successfully from the orbital wall with slightly increased force. The hook was then removed from the orbit under live fluoroscopy by twisting the Kocher in order to allow the hook to exit along the entrance trajectory to prevent iatrogenic injury (Figures 4(a) and 4(b)). No significant hemorrhage was appreciated from the puncture site after removal of the IOFB, and the wound was copiously irrigated with bacitracin-infused saline. Cerebrospinal fluid (CSF) leak was not appreciated.

**Figure 5:**
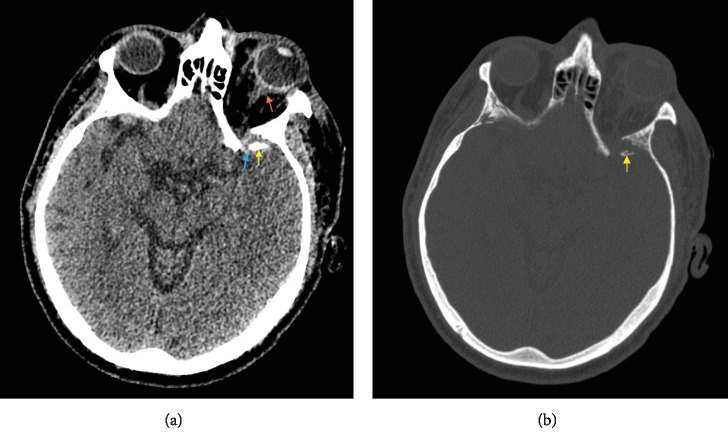
(a) Axial noncontrast CT head brain and (b) bone window obtained after foreign object removal demonstrating a left sphenoid wing comminuted fracture (yellow arrow) and associated small retrobulbar (orange arrow) and subarachnoid hemorrhage (blue arrow).

**Figure 6:**
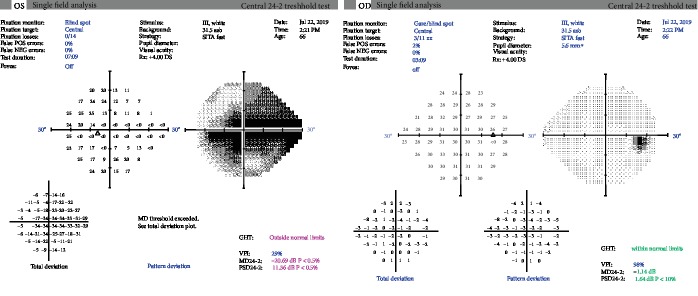
Humphrey 24-2 visual field. OD: full with no defect; OS: large central and cecocentral defect with superior and inferior nasal steps.

Postoperatively, he was taken for a conventional CT which showed a displaced comminuted fracture of the greater sphenoid wing, a small amount of retrobulbar hematoma, and a trace left anterior temporal contusion with adjacent subarachnoid hemorrhage (Figure 5). The optic nerve was not suspected to be injured by the fracture fragments as the fractures were lateral to the optic canal. The sphenoid wing fractures were assessed by the neurosurgery service, and no operative repair was indicated given the lack of CSF leak. He was admitted to intensive care unit for blood pressure control and frequent neurologic checks as a result of cerebral contusion and subarachnoid hemorrhage. A repeat CT scan six hours after his surgery was performed to rule out further hemorrhage. The patient underwent ophthalmologic examination daily, without further deterioration of the visual acuity or signs of retrobulbar hematoma or other neurological deficits. Three weeks after the initial injury, the patient had a persistent RAPD and no further improvement in vision. Follow-up vascular imaging two weeks after the incident in the form of CT angiography of the head ruled out delayed vascular injury, pseudoaneurysm, or carotid-cavernous fistula. A formal Humphrey visual field test was performed two weeks after the initial injury which showed a full visual field in the right eye and a central and cecocentral defect with superior and inferior nasal steps in the left eye (Figure 6). At 3 months, the patient's best-corrected Snellen vision improved to 20/30, with persistent RAPD in the left eye.

## 3. Discussion

Penetrating orbital injury with intracranial extension can potentially have a devastating prognosis. Furthermore, when a foreign body is retained in the orbit, management becomes even more complicated. The specific management is dependent on the structures injured as well as the intrinsic material of the foreign body, in addition to the size and the location of the foreign body [1, 3–5].

Most IOFB are categorized into organic or nonorganic material [6]. Organic IOFB warrant aggressive measures to achieve removal due to high risk of intraorbital infection [1, 7]. Nonorganic IOFB do not always require removal, especially if the IOFB is small and comprised of an inert metal. Nonorganic IOFB may be appropriately managed conservatively if they are thought to be in an inaccessible location which would lead to potential serious complications if surgical removal were to be performed. For example, a ferromagnetic metallic foreign body can remain harmless for years; however, they can cause serious ocular injuries when exposed to strong magnetic forces. This serves as a permanent contraindication for the patient to undergo magnetic resonance imaging (MRI) [6].

Strategizing an appropriate surgical approach for removal of an IOFB is incredibly important, as the wrong approach may result in significant morbidity or even mortality. If vascular injury is suspected, an anterior approach carries the risk of bleeding and death following removal of the foreign body due to sudden release of the tamponading effect of the foreign body over the injured vasculature [8]. If there is evidence of obvious violation of vasculature, a transcranial approach is often preferred, as it offers better control of the neurovascular anatomy.

In our case, performing a craniotomy prior to IOFB removal in order to directly visualize injured structures and to guide removal was considered. However, this was not pursued because the preoperative workup did not show dural laceration and neurologic or intracranial vascular injury. Durotomy would need to be performed in order to visualize the IOFB through the craniotomy which would create more opportunity for infection and CSF leak. A transorbital approach was selected, due to good visualization of neurovascular integrity, the mechanism of injury, the source of IOFB, and the reconstructed 3D image showing no violation of vascular structure. The low-velocity injury and blunt tip of the coat hook (Figure 3) made it less likely to cause dural or vascular laceration.

Visualization of the foreign object is the key to identifying the appropriate management. CT remains the ultimate cranial imaging modality for emergencies. CTs are obtained rapidly and allow for quick identification of hemorrhages and bony injuries with excellent detail. While the quality of soft tissue structures is far inferior to MRI, thin-cut CT does provide vital (and fast) assessment of the orbital integrity and may reveal other intracranial injuries such as hemorrhagic contusions and hematomas. This enabled rapid surgical planning and intervention. With the administration of contrast dye, CTA provides excellent vascular assessment. In fact, advancements in CTA technology are comparable to formal catheter angiography, and such can be performed on a much faster time scale without significant risk of stroke or arterial dissection. Catheter angiography via digital subtraction angiography (DSA) remains the gold standard of vascular integrity; although there is a small risk of stroke or vascular injury during catheterization, the images allow for selective visualization of vessels during the arterial and venous phase. Active hemorrhage or vascular injury can be diagnosed and treated via DSA, creating an incredibly powerful tool. Although MRI has many advantages to CT, it has limited applications in intracranial trauma assessment because of the use of powerful magnets, significant length of data acquisition, and overall limited availability in most hospitals on an emergent basis.

The decision of using high-dose intravenous steroid was deferred due to questionable intracranial involvement initially. Following the removal of IOFB, the decision of high-dose steroid was withheld due to more than eight hours passing since the incident of trauma. In addition, there are no convincing data that steroids provide any additional visual benefit over observation alone [9].

In our case, multidisciplinary careful planning was warranted. IR-guided removal allowed for better intraoperative visualization of orbitocranial structures and the new onset of hemorrhages. After the removal of the IOFB, if a large hemorrhage was appreciated on the open-cone CT scan, neurosurgery was prepared to perform a craniotomy for hemorrhage removal or CSF leak repair if necessary. If there was a sign of arterial injury with massive hemorrhage, IR was available to perform a diagnostic angiogram with possible embolization. Likewise, if there was a pseudoaneurysm or dissection, neurosurgery and/or IR remained available for potential stent, clip, or coil embolization. If there was a secondary globe rupture, ophthalmology was readily available for surgical repair.

Our case report demonstrates a very interesting presentation of an intraorbital foreign body. The management of which required multidisciplinary expertise to achieve the best clinical outcome. The nature of this injury was unusual, and the ball valve implantation of the hook into the orbit was remarkable. The patient was fortunate that the hook did not penetrate further and cause serious intracranial injuries. Good clinical and radiographic workup combined with surgical treatment allowed for a thorough yet expeditious successful management of this unique pathology.

## Abbreviations

IOFB: Intraorbital foreign body
ER: Emergency room
IR: Interventional radiology
EMS: Emergency medical service
GCS: Glasgow Coma Scale
OS: Oculus sinister
RAPD: Relative afferent pupillary defect
CT: Computed tomography
CTA: CT angiography
MRI: Magnetic resonance imaging
3D: Three-dimensional
CSF: Cerebrospinal fluid
DSA: Digital subtraction angiography.
